# Supplementary material for: The melanocortin action is biased toward protection from weight loss in mice
Source: Nat Commun. 2023 Apr 17;14:2200. doi: 10.1038/s41467-023-37912-z (PMC10110624; doi:10.1038/s41467-023-37912-z)
Supplement: Supplementary file 1 — Supplementary Information [file 41467_2023_37912_MOESM1_ESM.pdf]

Supplementary Figures

The Melanocortin Action Is Biased Toward Body Weight Gain

Hongli Li, Yuanzhong Xu, Yanyan Jiang, Zhiying Jiang, Joshua Otiz-Guzman, Jessie C. Morrill, Jing Cai, Zhengmei Mao, Yong Xu, Benjamin R. Arenkiel,

Cheng Huang and Qingchun Tong

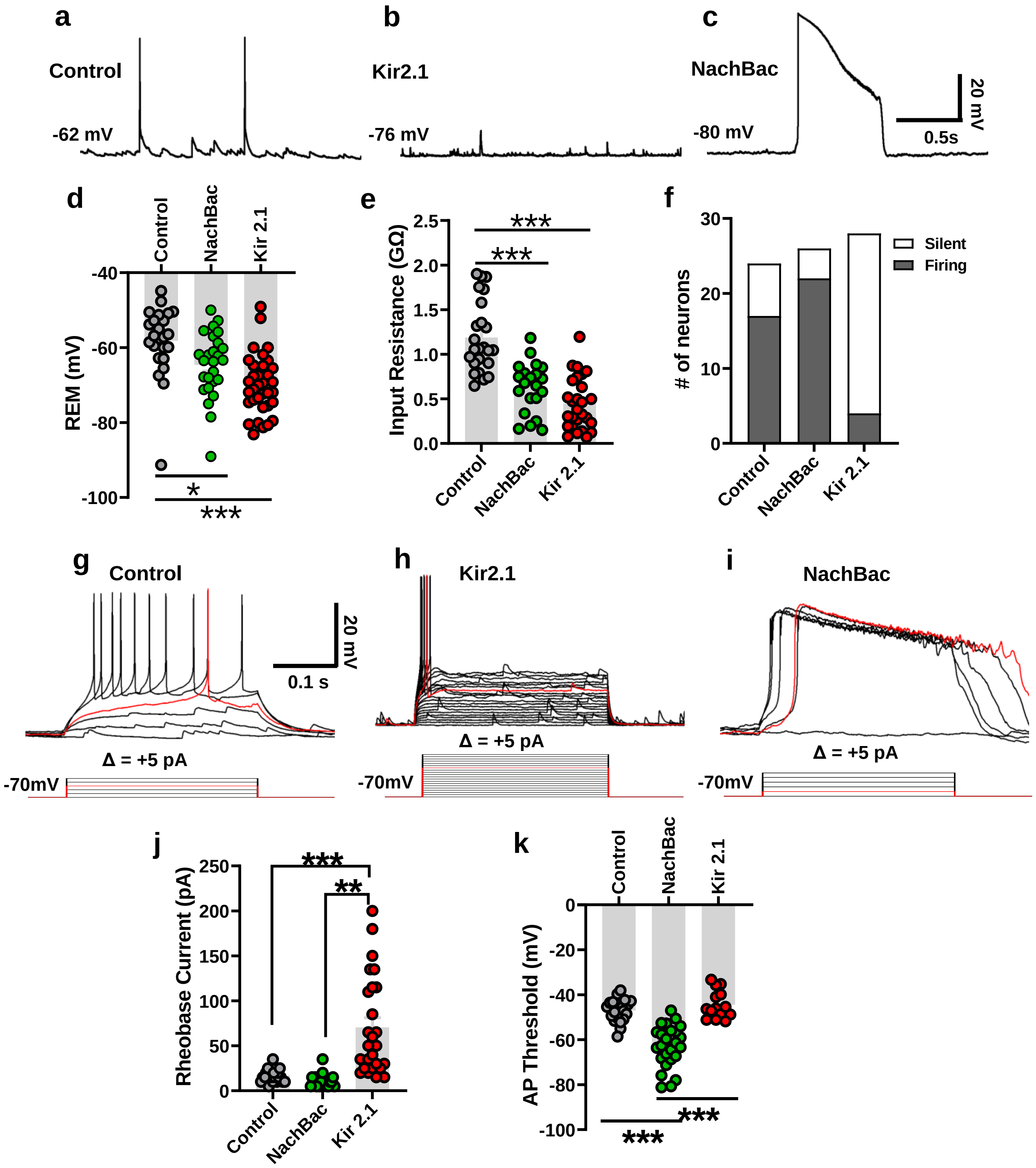

Supplementary Fig. 1. Changes in POMC neuron excitability with the expression of NachBac or Kir2.1. Brain slices from POMC-Cre mice with POMC neurons expressing AAV-EF1a-flex-EGFP-P2A-mNachBac, AAV-EF1a-DIO-Kir2.1-P2A-dTomato or control vectors were used for patch-clamp recording. (**a-c**) Representative traces showing resting membrane potential (REM) and spontaneous action potential (AP) firing in control (**a**), NachBac (**b**) and Kir2.1 neurons (**c**). (**d-f**) Comparison in REM (**d**, n = 24 for control, 26 for NachBac and 34 for Kir2.1, one-way ANOVA followed by Turkey's multiple comparisons, F (2, 81) = 13.25; Control vs. NachBac * p = 0.0264, Control vs. Kir2.1 *** p < 0.001), input resistance (**e**, n = 24 for control, 21 for NachBac and 27 for Kir2.1, one-way ANOVA followed by Turkey's multiple comparisons, F (2, 69) = 34.11; Control vs. NachBac *** p < 0.001, Control vs. Kir2.1 *** p < 0.001) and number of all recording neurons with spontaneous AP firing (**f**). (**g-j**) Representative traces showing elicitation of AP firing by injecting step-wise depolarizing currents in controls (**g**), Kir2.1 (**h**) and NachBac neurons (**i**). The traces labelled in red representing the rheobase currents. (**j-k**) Statistical comparisons of rheobase (**j**, n = 22 for control, 19 for NachBac and 26 for Kir2.1, one-way ANOVA followed by Turkey's multiple comparisons, F (2, 64) = 21.26; Control vs. Kir2.1 *** p < 0.001, NachBac vs. Kir2.1 *** p < 0.001) and AP firing threshold (**k**, n = 15 for control, 25 for NachBac and 14 for Kir2.1, one-way ANOVA followed by Turkey's multiple comparisons, F (2, 59) = 41.17; Control vs. NachBac *** p < 0.001, NachBac vs. Kir2.1 *** p < 0.001). All data were presented as mean ± SEM. Source data are provided as a Source Data file.

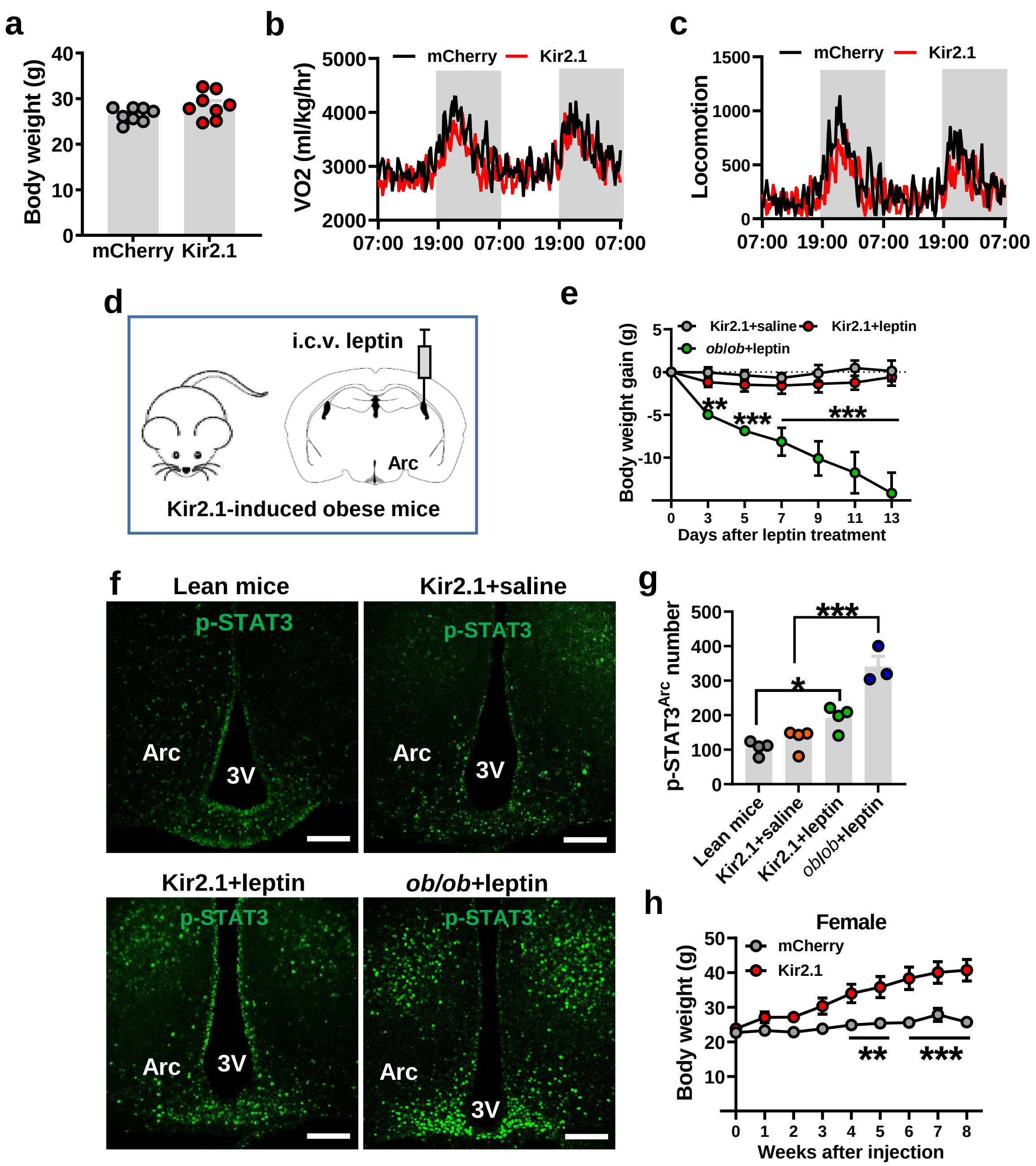

Supplementary Fig. 2. Kir2.1 expression in POMC neurons led to obese phenotype. (**a-c**) Body weight comparison between POMC-Cre mice with Kir2.1 or control mCherry viral injection to the Arc when subjected to CLAMS measurements (**a**, n = 8/each, unpaired Student's *t* tests, t = 1.750, df = 14; $p = 0.102$), and their actual traces of O_2 consumption (**b**) and locomotion patterns (**c**) during the 2-day measurement period. (**d-f**) Male POMC-Cre mice with Kir2.1- induced obesity were treated with i.c.v. infusion of leptin for 2 weeks (**d**), and measured body weight (**e**, n = 4 for Kir2.1+ saline, 4 for Kir2.1+ leptin, and 3 for *ob/ob*+leptin, two-way ANOVA, F (1.301, 10.41) = 23.05; Kir2.1+saline vs. *ob/ob*+leptin ** p =0.0027 at day 3, *** $p = 0.002$ at day 5 and *** $p < 0.0001$ at days 7-13), representative pictures (f) and quantification of p-STAT3 expression in the Arc (**g**, n = 4 for lean, Kir2.1+saline and Kir2.1+ leptin groups, n = 3 for *ob/ob*+leptin group, lean vs. Kir2.1+leptin * p = 0.0221, *ob/ob*+leptin *vs* Kir2.1+ leptin *** $p = 0.008$, *ob/ob*+leptin vs. lean and Kir2.1+ saline *** $p < 0.001$). Arc: arcuate nucleus; 3v: third ventricle. Scale bars: 50 µm. (**h**) Body weight growth curves in female POMC-Cre mice following Kir2.1 viral delivery to the Arc (n = 5/each, two-way ANOVA, F (8, 72) = 8.848; mCherry vs.Kir2.1 ** $p = 0.0075$ at week 4, ** $p = 0.0014$ at week 5, and *** $p < 0.001$ at weeks 6-8). All data were presented as mean ± SEM. Source data are provided as a Source Data file.

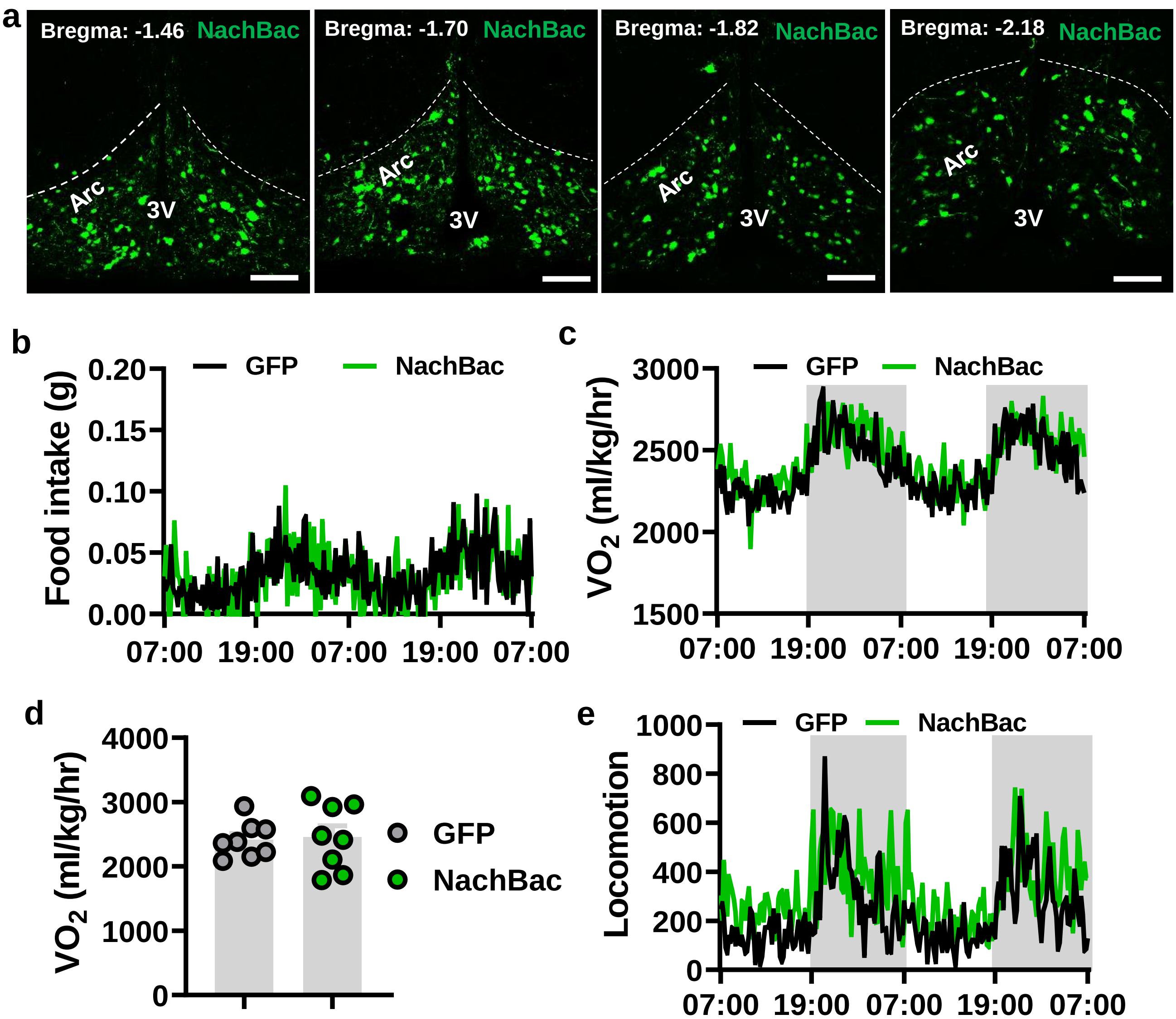

Supplementary Fig. 3. Phenotypes of mice with POMC neurons expressing NachBac. (**a**) Representative images showing NachBac expression in Arc POMC neurons in a series of rostral to caudal brain sections. Arc: arcuate nucleus; 3V: third ventricle. Scale bars: 50 µm. (**b-e**) CLAMS measurements of male POMC-Cre mice with either NachBac or control GFP viral injection to the Arc and fed with HFD, and results showing representative traces of feeding (**b**), O_2 consumption (**c** and **d**, n = 8/each, 2-tailed unpaired Student's *t* tests; t = 0.1974, df = 14, *p* = 0.8643), and locomotion (**e**) patterns of the two groups. All data were presented as mean ± SEM. Source data are provided as a Source Data file.

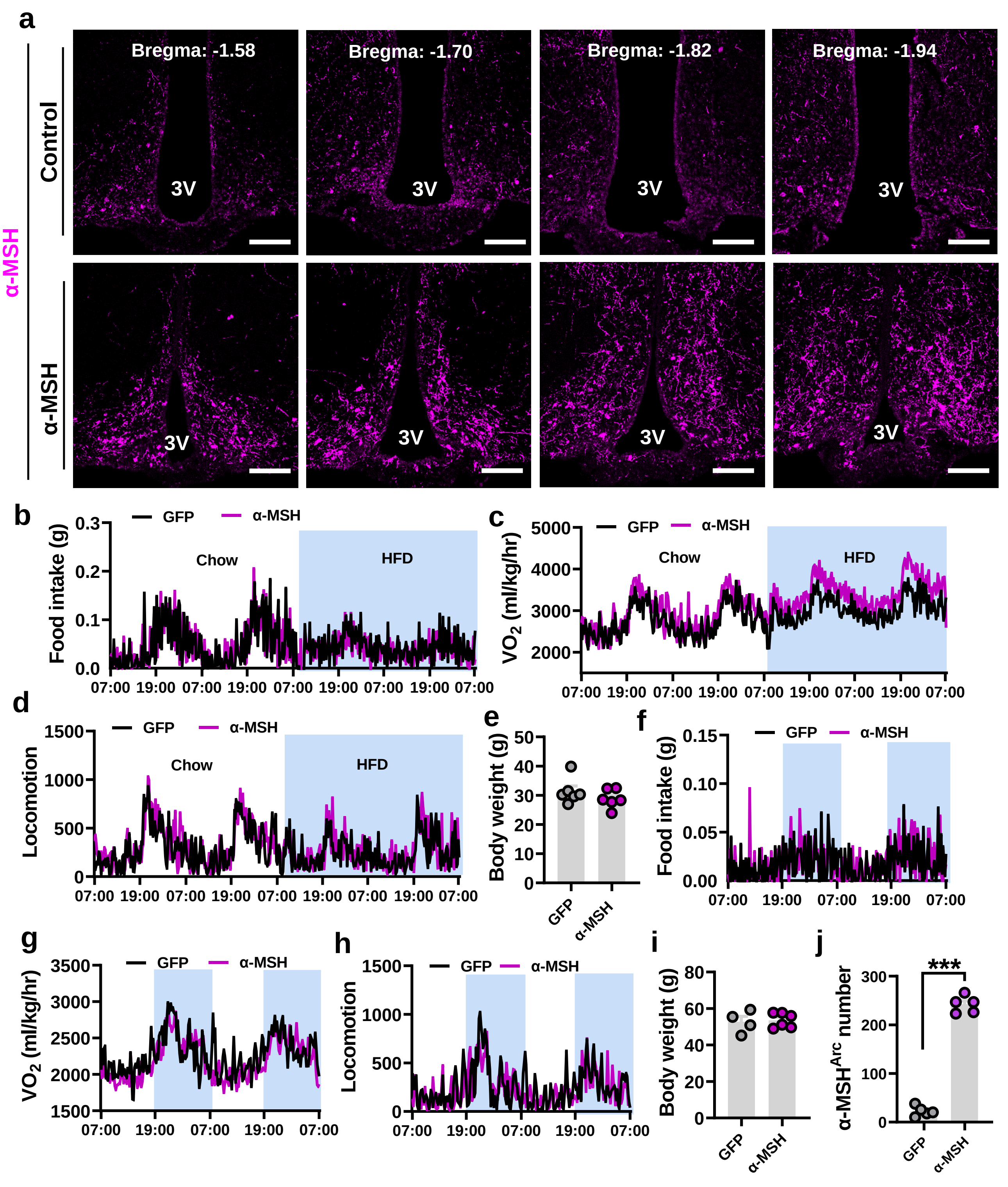

Supplementary Fig. 4. Phenotypes of mice with POMC neurons overexpressing α-MSH. (**a**) Representative images showing α-MSH expression pattern in POMC neurons of POMC-Cre mice with control (top panels) or α-MSH viral injection to the Arc. 3V: third ventricle. Scale bars: 50 μm. (**b-e**) CLAMS measurements of male POMC-Cre mice that received either control or α-MSH viral injection to the Arc during a transition from chow to HFD, and results showing representative traces of feeding (**b**), O_2 consumption (**c**), and locomotion (**d**) patterns, and body weight comparison between the 2 groups when the measurement was started (**e**, n = 6/each, 2-tailed unpaired Student's *t* tests; t = 1.143, df = 10, *p* = 0.2795). (**f-i**) CLAMS measurements of male POMC-Cre mice that were firstly subjected to HFD feeding for obesity development and then receive either control or α-MSH viral injection to the Arc, and results showing feeding (**f**), O_2 consumption (g) and locomotion (**h**) patterns, and body weight comparison between the 2 groups when measurement was started (**i**, n = 4 for GFP and 6 for α-MSH, 2-tailed unpaired Student's *t* tests; t = 0.2536, df = 8, *p* = 0.8062). (**j**) Quantification of α-MSH+ neurons in the Arc following the injection of control GFP or α-MSH viral vectors in POMC neurons of male mice (n = 5/each, 2-tailed unpaired Student's *t* tests; t = 23.95, df = 8, *** *p* <0.0001) . All data were presented as mean ± SEM. Source data are provided as a Source Data file.

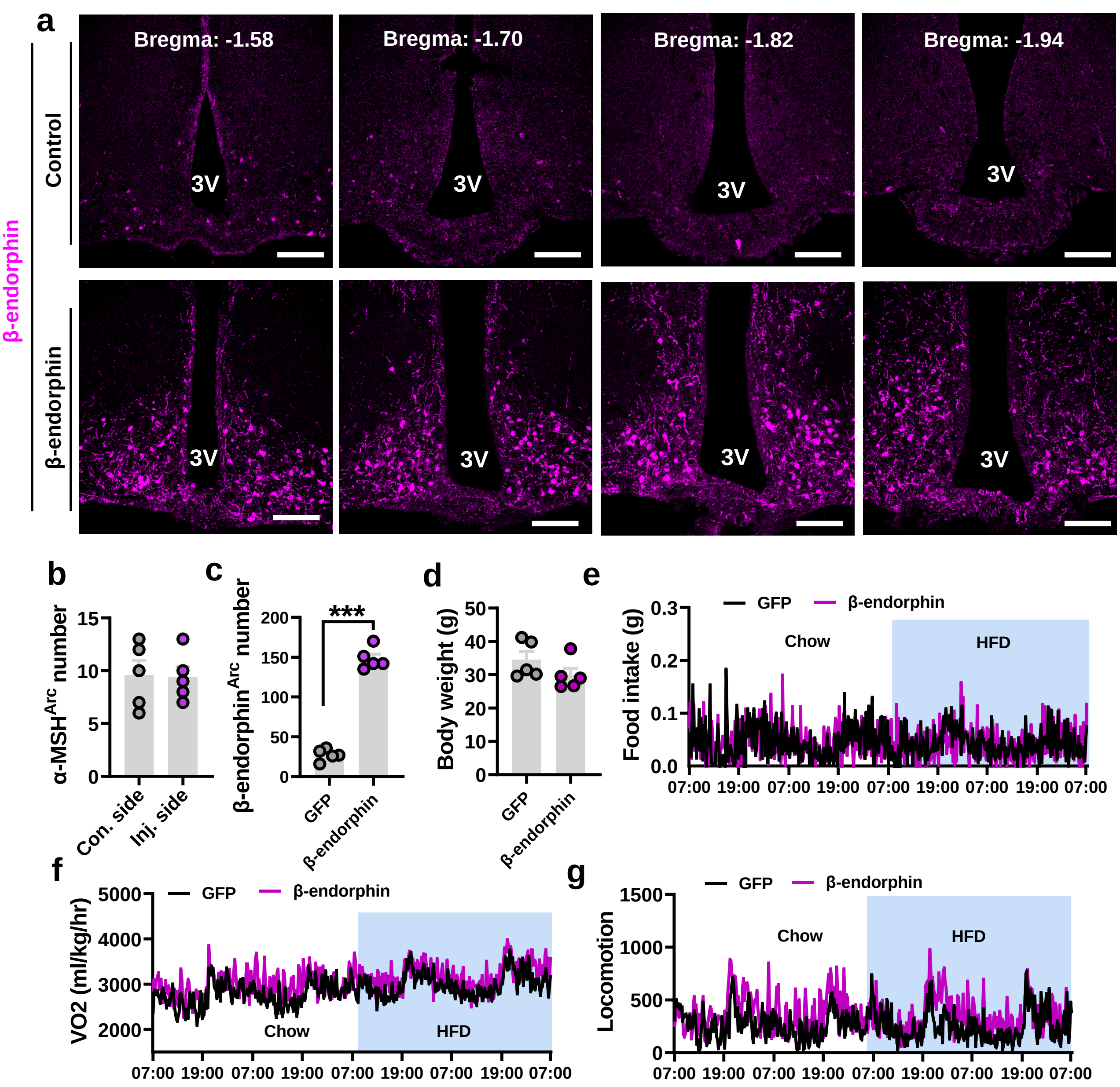

Supplementary Fig. 5. Phenotypes of mice with POMC neurons overexpressing β-endorphin. (**a**) Representative images showing β-endorphin expression patterns in POMC neurons of POMC-Cre mice with control (top panels) or β-endorphin viral injection to the Arc. 3V: the third ventricle; Scale bars: 50 µm. (**b**) Quantification of β-endorphin+ cell number in the Arc following the unilateral injection of GFP or β-endorphin viral vectors in male mice (Related to **Fig. 4c**, n = 5/each, 2-tailed paired Student's *t* tests; t = 0.1166, df = 4, *p* = 0.9128). (**c**) Quantification of β-endorphin+ cell number in the Arc following the bilateral injection of GFP or β-endorphin viral vectors in male mice (n = 5/each, 2-tailed unpaired Student's *t* tests; t = 17.40, df = 8, ****p* < 0.001). (**d-g**) CLAMS measurements of male POMC-Cre mice that received either control or β-endorphin viral injection to the bilateral Arc during the chow to HFD transition, and results showing body weight comparison between the 2 groups when the measurement was started (**d**, n = 5/each, 2-tailed unpaired Student's *t* tests; t = 1.408, df = 8, *p* = 0.1967) and their feeding (**e**), O_2 consumption (**f**) and locomotion (**g**) patterns during the test. All data were presented as mean ± SEM. Source data are provided as a Source Data file.

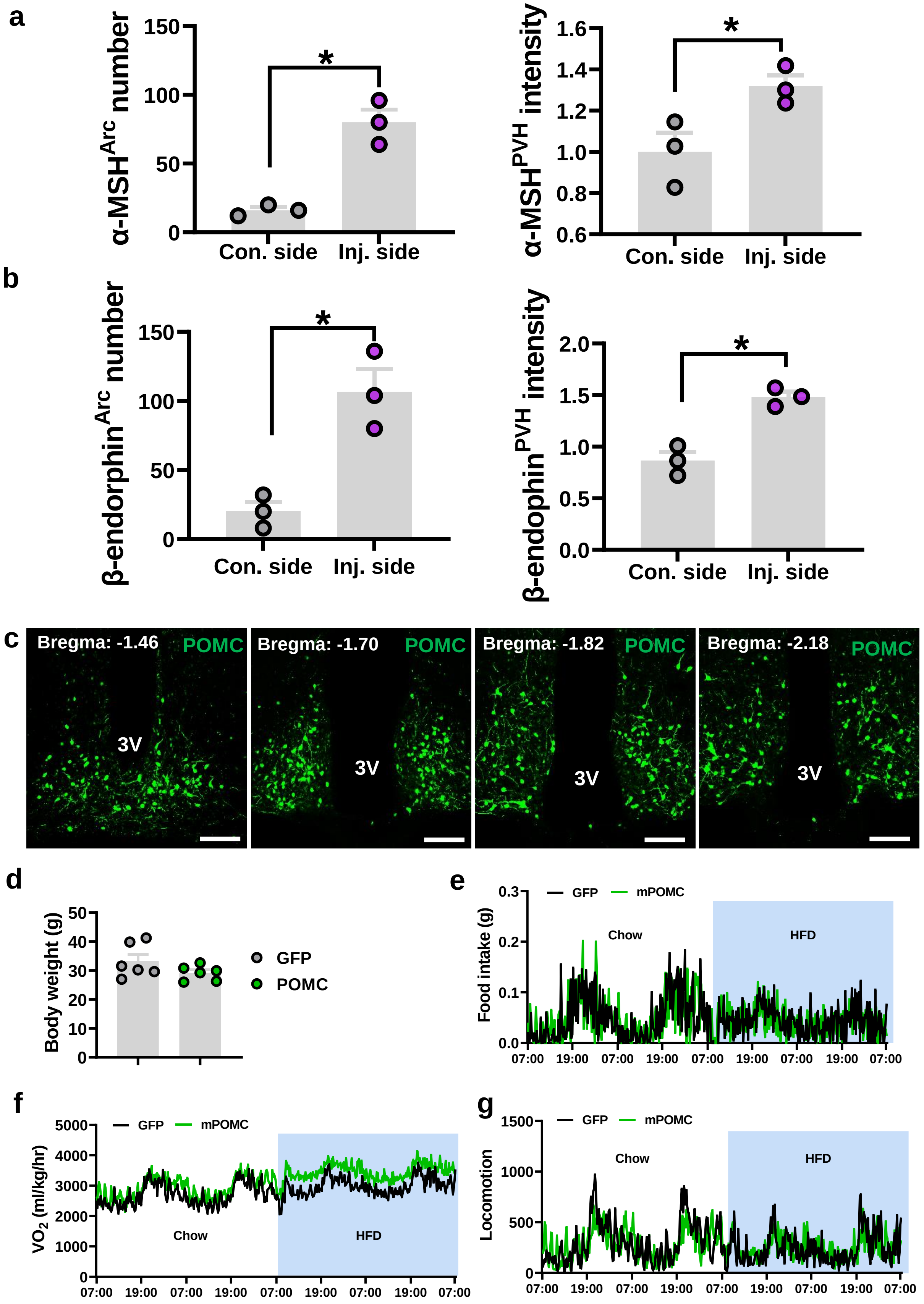

a
α-MSH^Arc number
150
100
50
0
*
Con. side
Inj. side
α-MSH^PVH intensity
1.6
1.4
1.2
1.0
0.8
0.6
*
Con. side
Inj. side
b
β-endorphin^Arc number
150
100
50
0
*
Con. side
Inj. side
β-endophin^PVH intensity
2.0
1.5
1.0
0.5
0.0
*
Con. side
Inj. side
c
Bregma: -1.46
POMC
3V
Bregma: -1.70
POMC
3V
Bregma: -1.82
POMC
3V
Bregma: -2.18
POMC
3V
d
Body weight (g)
50
40
30
20
10
0
GFP
POMC
e
Food intake (g)
0.3
0.2
0.1
0.0
GFP
mPOMC
Chow
HFD
07:00
19:00
07:00
19:00
07:00
19:00
07:00
19:00
07:00
f
VO2 (ml/kg/hr)
5000
4000
3000
2000
1000
0
GFP
mPOMC
Chow
HFD
07:00
19:00
07:00
19:00
07:00
19:00
07:00
19:00
07:00
g
Locomotion
1500
1000
500
0
GFP
mPOMC
Chow
HFD
07:00
19:00
07:00
19:00
07:00
19:00
07:00
19:00
07:00

Supplementary Fig. 6. Phenotypes of mice with POMC neurons overexpressing POMC. (**a** and **b**) Quantification of α-MSH (n = 3/each, 2-tailed paired Student's *t* tests; * *p* = 0.0310 for left panel, * p = 0.0198 for right panel) and β-endorphin (n = 3/each, 2-tailed paired Student's *t* tests; * *p* = 0.0275 for left panel, * *p* = 0.0452 for right panel) expression in the Arc and PVH with mPOMC overexpression in POMC neurons of the unilateral Arc in male mice. (**c**) Representative images showing POMC viral vector expression in Arc POMC neurons in a series of rostral to caudal brain sections. 3V: the third ventricle; Scale bars: 50 µm. (**b-d**) CLAMS measurements of male POMC-Cre mice that received either control (GFP) or POMC viral injection to the Arc during the chow to HFD transition, and results showing no body weight difference when the measurement was started (**d**, n = 6/each, 2-tailed unpaired Student's *t* tests; t=1.566, df=10, *p* = 0.4185) and representative traces of feeding (**e**), O_2 consumption (**f**) and locomotion (**g**) patterns of the 2 groups. All data were presented as mean ± SEM. Source data are provided as a Source Data file.

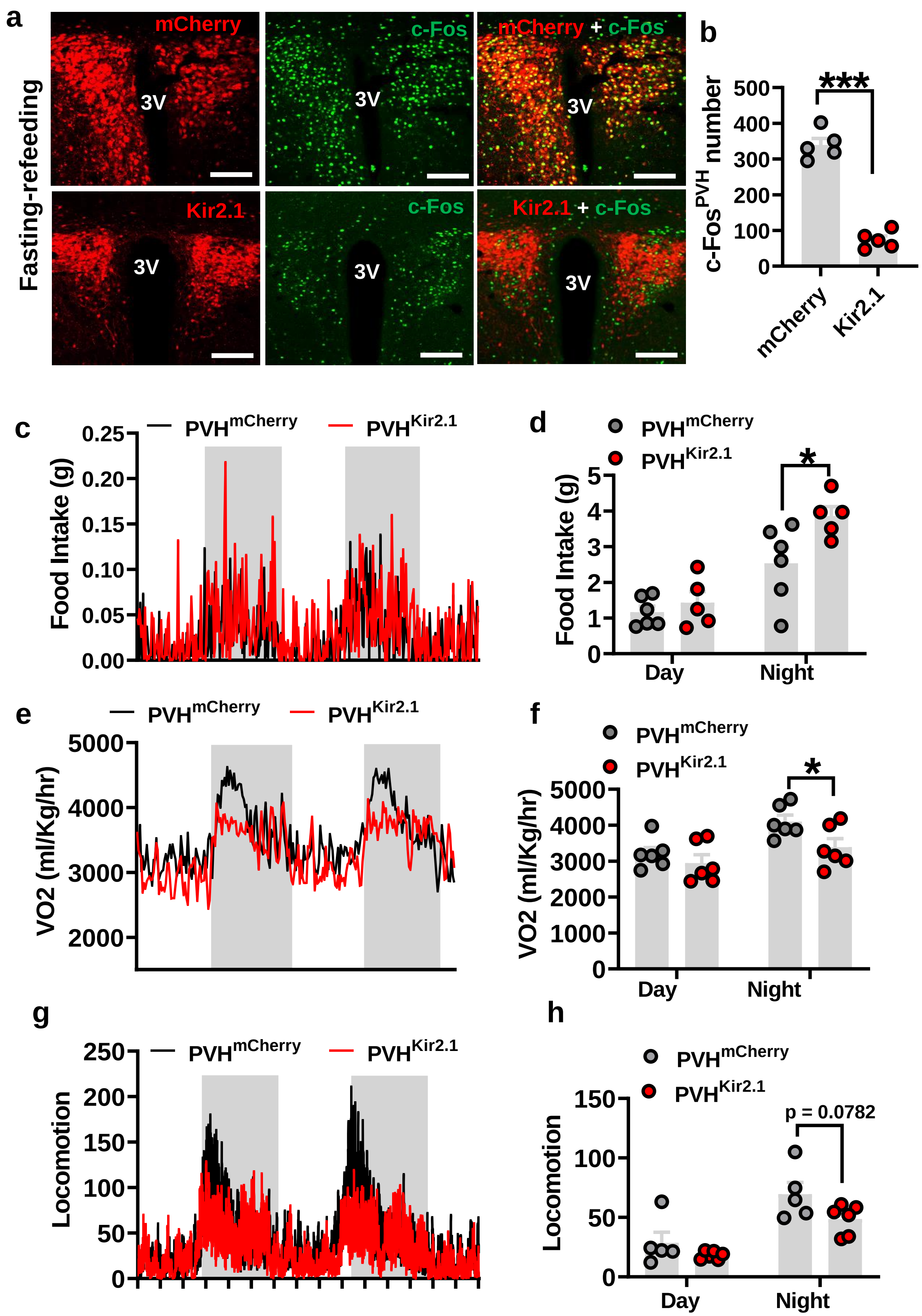

a
Fasting-refeeding
mCherry
c-Fos
mCherry + c-Fos
Kir2.1
Kir2.1 + c-Fos
3V
b
c-Fos^PVH number
***
mCherry
Kir2.1
c
Food Intake (g)
PVH^mCherry
PVH^Kir2.1
d
Day
Night
e
VO2 (ml/Kg/hr)
f
g
Locomotion
h
p = 0.0782

Supplementary Fig. 7. Phenotypes of mice with PVH MC4R neurons expressing Kir2.1. (**a**) Representative images showing the effect of Kir2.1 expression on c-Fos induction in PVH MC4R neurons during fast-refeeding. An obvious reduction of c-Fos expression was showed in the PVH of Kir2.1-injected mice compared to controls. 3V: the third ventricle; Scale bars: 50 µm. (**b**) Quantification of c-Fos expression in the PVH of male MC4R-Cre mice with either control or Kir2.1 viral injection to the PVH after overnight fasting and followed by 4 hours refeeding (n = 5 each group; 2-tailed unpaired Student's *t* tests, t = 12.59, df = 8 *** $p < 0.0001$) (**c-h**) Male MC4R-Cre mice (8-10 weeks old) that received either control or Kir2.1 viral injection to the PVH were used for CLAMS measurements. The measurement was conducted during the first week after viral delivery when there was no body weight difference between the 2 groups. (**c-d**) Feeding patterns (**c**) and statistical comparisons in food intake during day and night periods (**d**, n = 6 for mCherry group and 5 for Kir2.1 group, 2-way ANOVA followed by Šídák's multiple comparisons; F (1, 18) = 35.75, mCherry vs. Kir2.1 p = 0.8136 for day time, * p = 0.0171 for night time). (**e-f**) O_2 consumption patterns (e) and statistical comparisons in O_2 consumption between the 2 groups during day and night periods (**f**, n = 6 each group, 2-way ANOVA followed by Šídák's multiple comparisons; F (1, 20) = 10.33, mCherry vs. Kir2.1 p = 0.6175 for day time, p = 0.0485 for night time). (**g-h**) Locomotion patterns (g) and statistical comparisons in beam breaks between the 2 groups during day and night periods (**h**, n = 5 for mCherry group and 6 for Kir2.1 group, 2-way ANOVA followed by Šídák's multiple comparisons; F (1, 18) = 28.51, mCherry vs. Kir2.1 p = 0.4887 for day time, p = 0.0782 for night time). All data were presented as mean ± SEM. Source data are provided as a Source Data file.

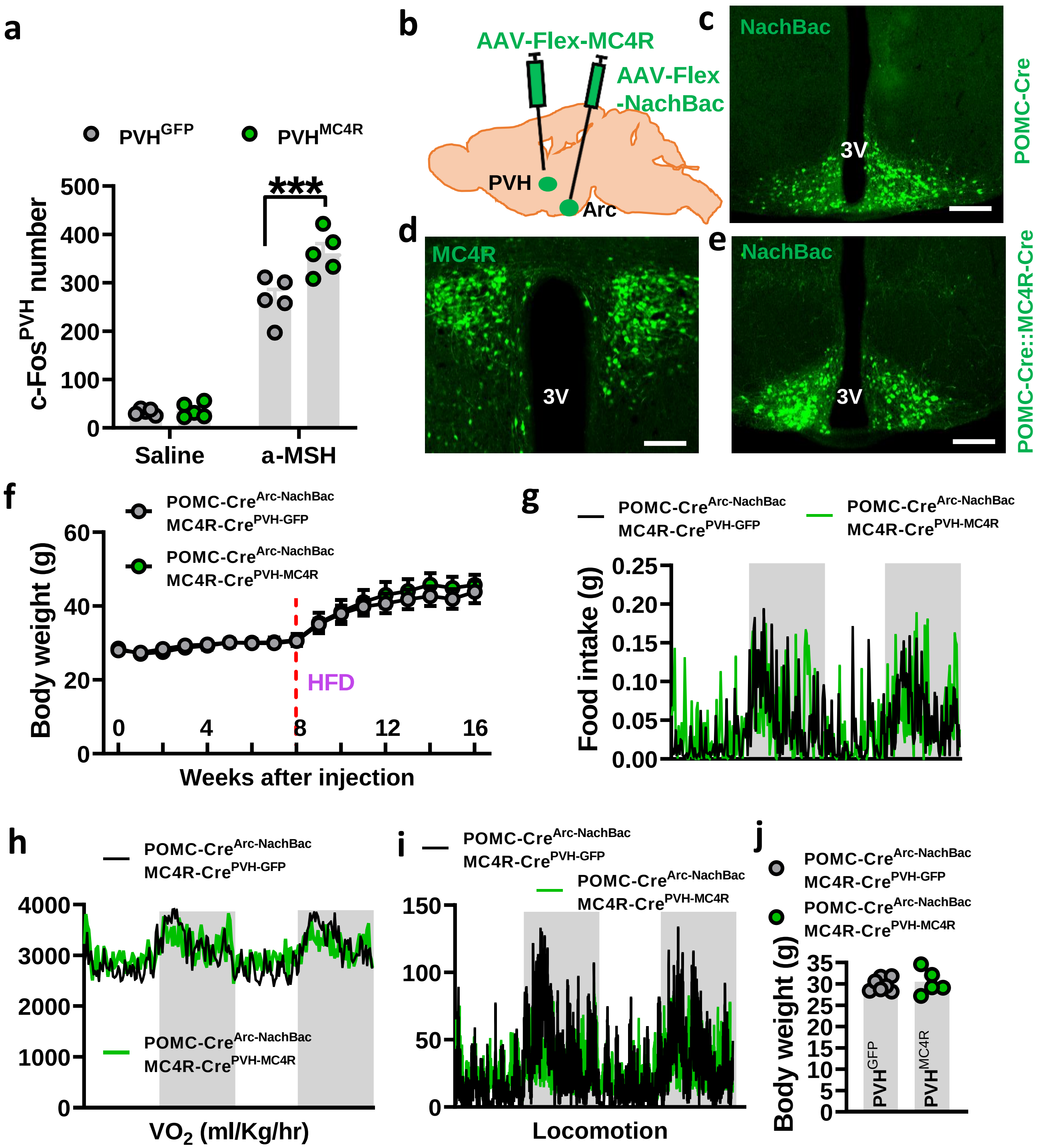

Supplementary Fig. 8. Mice with concurrent activation of POMC neurons and overexpression of MC4Rs in PVH^{MC4R} neurons showed no difference in body weight. (**a**) Quantification of c-Fos expression in the PVH of mice with MC4Rsoverexpression in PVH^{MC4R} neurons following i.c.v. α-MSH (4μg) injection (n = 5 each group, two-way ANOVA by Šídák's multiple comparisons; F (1, 16) = 366.8, PVH^{GFP} vs. PVH^{MC4R} p = 0.9852 for saline, *** p = 0.0006 for α-MSH). (**b**) Diagram showing the injection strategy in 7-8 week-old POMC-Cre::MC4R-Cre mice, in which the AAV-Flex-NachBac virus was delivered to the bilateral Arc and AAV-Flex-MC4R-P2A-GFP (or AAV-Flex-GFP as control) was delivered to the bilateral PVH. (**c**) Representative NachBac expression pattern in the Arc of control POMC-Cre::MC4R-Cre mice. (**d-e**) Representative expression pattern of the MC4R viral vectors in the PVH (d) and NachBac viral vectors in the Arc (**e**) in POMC-Cre::MC4R-Cre mice. 3V: third ventricle. Scale bars: 100 μm in (**c**) and (**e**), 50 μm in (**d**). (**f**) Body weight curves of the 2 groups during the 8 weeks measurement of chow feeding and the following 8 weeks measurement of HFD feeding after viral delivery on chow diet (n = 6/each, 2-way ANOVA followed by Šídák's multiple comparisons; F (16, 170) = 21.56, p > 0.99 for all time points). (**g-i**) CLAMS measurements of male POMC-Cre::MC4R-Cre mice that received either Arc- NachBac and PVH-GFP (control) injection or Arc-NachBac and PVH-MC4R injection, and results showing representative traces of feeding (**g**), O_2 consumption (**h**) and locomotion (**i**) patterns of the 2 groups when no body weight difference was recorded (**j**, n = 7 for PVH-GFP group and 5 for PVH-MC4R group, 2-tailed unpaired Student's t tests, t = 0.4657, df = 10, p = 0.1573). All data were presented as mean ± SEM. Source data are provided as a Source Data file.
