# Supplementary material for: The melanocortin action is biased toward protection from weight loss in mice
Source: Nat Commun. 2023 Apr 17;14:2200. doi: 10.1038/s41467-023-37912-z (PMC10110624; doi:10.1038/s41467-023-37912-z)
Supplement: Supplementary file 3 — Reporting Summary [file 41467_2023_37912_MOESM3_ESM.pdf]

nature portfolio

Corresponding author(s): NCOMMS-22-41785A-Z

Last updated by author(s): Mar 23, 2023

Reporting Summary

Nature Portfolio wishes to improve the reproducibility of the work that we publish. This form provides structure for consistency and transparency in reporting. For further information on Nature Portfolio policies, see our Editorial Policies and the Editorial Policy Checklist.

Statistics

For all statistical analyses, confirm that the following items are present in the figure legend, table legend, main text, or Methods section.

n/a	Confirmed	
☐	☒	The exact sample size (*n*) for each experimental group/condition, given as a discrete number and unit of measurement
☐	☒	A statement on whether measurements were taken from distinct samples or whether the same sample was measured repeatedly
☐	☒	The statistical test(s) used AND whether they are one- or two-sided<br>*Only common tests should be described solely by name; describe more complex techniques in the Methods section.*
☐	☒	A description of all covariates tested
☐	☒	A description of any assumptions or corrections, such as tests of normality and adjustment for multiple comparisons
☐	☒	A full description of the statistical parameters including central tendency (e.g. means) or other basic estimates (e.g. regression coefficient) AND variation (e.g. standard deviation) or associated estimates of uncertainty (e.g. confidence intervals)
☐	☒	For null hypothesis testing, the test statistic (e.g. *F*, *t*, *r*) with confidence intervals, effect sizes, degrees of freedom and *P* value noted<br>*Give P values as exact values whenever suitable.*
☒	☐	For Bayesian analysis, information on the choice of priors and Markov chain Monte Carlo settings
☒	☐	For hierarchical and complex designs, identification of the appropriate level for tests and full reporting of outcomes
☒	☐	Estimates of effect sizes (e.g. Cohen's *d*, Pearson's *r*), indicating how they were calculated

Our web collection on statistics for biologists contains articles on many of the points above.

Software and code

Policy information about availability of computer code

Data collection	Microsoft Excel 2020, GraphPad Prism 9 and pCLAMP10
Data analysis	GraphPad Prism 9 and Adobe Photoshop CS6.

For manuscripts utilizing custom algorithms or software that are central to the research but not yet described in published literature, software must be made available to editors and reviewers. We strongly encourage code deposition in a community repository (e.g. GitHub). See the Nature Portfolio guidelines for submitting code & software for further information.

Data

Policy information about availability of data

All manuscripts must include a data availability statement. This statement should provide the following information, where applicable:

- Accession codes, unique identifiers, or web links for publicly available datasets
- A description of any restrictions on data availability
- For clinical datasets or third party data, please ensure that the statement adheres to our policy

We have provides raw data on those figures that are not provided with individual data points. We have also provided all information on statistical analysis including effects size, degree of freedom and exact p values.

Human research participants

Policy information about studies involving human research participants and Sex and Gender in Research.

Reporting on sex and gender	*Use the terms sex (biological attribute) and gender (shaped by social and cultural circumstances) carefully in order to avoid confusing both terms. Indicate if findings apply to only one sex or gender; describe whether sex and gender were considered in study design whether sex and/or gender was determined based on self-reporting or assigned and methods used. Provide in the source data disaggregated sex and gender data where this information has been collected, and consent has been obtained for sharing of individual-level data; provide overall numbers in this Reporting Summary. Please state if this information has not been collected. Report sex- and gender-based analyses where performed, justify reasons for lack of sex- and gender-based analysis.*
Population characteristics	*Describe the covariate-relevant population characteristics of the human research participants (e.g. age, genotypic information, past and current diagnosis and treatment categories). If you filled out the behavioural & social sciences study design questions and have nothing to add here, write "See above."*
Recruitment	*Describe how participants were recruited. Outline any potential self-selection bias or other biases that may be present and how these are likely to impact results.*
Ethics oversight	*Identify the organization(s) that approved the study protocol.*

Note that full information on the approval of the study protocol must also be provided in the manuscript.

Field-specific reporting

Please select the one below that is the best fit for your research. If you are not sure, read the appropriate sections before making your selection.

☒ Life sciences ☐ Behavioural & social sciences ☐ Ecological, evolutionary & environmental sciences

For a reference copy of the document with all sections, see nature.com/documents/nr-reporting-summary-flat.pdf

Life sciences study design

All studies must disclose on these points even when the disclosure is negative.

Sample size	Sample size (n=6-8) was predetermined base literature and our own experience in body weight studies. Given the dramatic difference in body weight observed in most of our studies, some of the study groups used n> or = 5. For a few control experiments such as body weight response of ob/ob mice to leptin administration, which has been well demonstrated in the literature as well as the dramatic effects of leptin in reducing obesity, we used n=3/group. For electrophysiologic studies, we used neuron number n=11 or 12 for leptin effects on neurons and n=15 or 19 for NachBac effects and n=16 or 21 for Kir2.1 effects. These neurons were from 3-4 animals each group. These numbers are within the ranges of similar studies in the related literature. Details were described in Figure Legends.
Data exclusions	Most of data collected was used for analysis. The exception is that, after posthoc analysis on AAV vector delivery, those with evidence of missed injections, i.e. not sufficient number neurons showing viral expression, were excluded for body weight analysis.
Replication	Due to long time required for each stereotaxic surgery, study group subjects of each group were performed during different days with differnet experiments. Results from these different experiments were comparable within the same group and combined.
Randomization	All animals with the same genotype were randomly divided into control or experimental groups.
Blinding	No blinding procedure was implemented. However, the researcher who measured body weight was not aware of the mouse grouping information.

Reporting for specific materials, systems and methods

We require information from authors about some types of materials, experimental systems and methods used in many studies. Here, indicate whether each material, system or method listed is relevant to your study. If you are not sure if a list item applies to your research, read the appropriate section before selecting a response.

Materials & experimental systems

n/a	Involved in the study
☐	☒ Antibodies
☒	☐ Eukaryotic cell lines
☒	☐ Palaeontology and archaeology
☐	☒ Animals and other organisms
☒	☐ Clinical data
☒	☐ Dual use research of concern

Methods

n/a	Involved in the study
☒	☐ ChIP-seq
☒	☐ Flow cytometry
☒	☐ MRI-based neuroimaging

Antibodies

Antibodies used

. Rabbit Anti-c-Fos Antibody; Millipore; ABE457,Species Reactivity:H, R; LOT# 2552627
2. Rabbit anti Phospho-Stat3 (Tyr705) (D3A7) XP Rabbit mAb;Cell signaling technology;#9145; Reactivity:H M R Mk; LOT # 18
3. Rabbit α-MSH (1:1000), #H-043-01, Phoenix Pharmaceuticals, CA, USA)
4. β-endorphin (1:1000, #H-022-33, Phoenix Pharmaceuticals, CA.
5. AlexaFluor 488 conjugated donkey anti-rabbit IgGs; Jackson ImmunoResearch Laboratories; LOT # 110898

Validation

All antibodies have been used and verified in the literature, and also verified by our own results with expected changes in control animals.

Animals and other research organisms

Policy information about studies involving animals; ARRIVE guidelines recommended for reporting animal research, and Sex and Gender in Research

Laboratory animals

All mice were housed with ad libitum access to water and food in a temperature controlled room (21°C-22°C) with a 12:12 hour light-dark cycle. Animal care and procedures were approved by the Animal Welfare Committee of The University of Texas Health Science Center at Houston. POMC-Cre or Mc4R-Cre mice were bred to Ai9 reporter to get generate respective reporter mice for electrophysiological recording, assessing viral delivery quality or colocalization with c-Fos immunohistochemistry. Ob/ob mice were verified in this study. These animals are on mixed background except for ob/ob mice, which were originally on the C57 background but bred with POMC-Cre mice. All mice were used from 7 weeks old to 20 weeks old of age Most of studies used males and the Kir2.1 effects were also performed in females.

Wild animals

This study didn't involve wild animals.

Reporting on sex

Most studies were conducted on males but some of key results, e.g. body weight, were also reproduced in females.

Field-collected samples

This study didn't involve samples collected from field.

Ethics oversight

All animal studies and associated research protocols are included in the animal protocols 21-111 and 21-155 approved by Animal Welfare Committee of UTHealth.

Note that full information on the approval of the study protocol must also be provided in the manuscript.
